# Generic Pharmaceuticals as a Source of Diuretic Contamination in Athletes Subject to Sport Drug Testing

**DOI:** 10.3389/fspor.2021.692244

**Published:** 2021-11-18

**Authors:** Amy Eichner, Laura A. Lewis, Bridget Leonard, Ryan M. Van Wagoner, Daniel Eichner, Matthew N. Fedoruk

**Affiliations:** ^1^United States Anti-Doping Agency, Colorado Springs, CO, United States; ^2^Sports Medicine and Research Testing Laboratory, Salt Lake City, UT, United States

**Keywords:** prescription medication, anti-doping, rule violation, minimum reporting level, adverse analytical finding

## Abstract

This paper describes nine instances of positive anti-doping tests that could be accounted for by the use of permitted generic prescription drugs contaminated with diuretics, which are prohibited in sport at all times under the WADA Prohibited List. The contamination levels found in the medications are reported and were below FDA limits for manufacturers that are based primarily on safety considerations. These cases demonstrate that great care must be taken to identify the source of low-level anti-doping positives for diuretics reported by WADA-accredited laboratories, and possibly other prohibited substances as well, in order to avoid sanctioning innocent athletes. An evaluation of the cases in this paper supports an approach which establishes a laboratory minimum reporting level (MRL) for diuretics found most commonly in medications. A global consensus after extensive review of similar anti-doping cases has resulted in implementation of a recently announced solution regarding potential diuretic contamination cases.

## Introduction

Sport drug testing is the backbone of the anti-doping movement and the fight for clean sport. Athlete's samples are tested in appropriately accredited laboratories according to international consensus standards published by the World Anti-Doing Agency. For the vast majority of substances on the WADA Prohibited List, any amount of the substance detected in the sample is regarded as a “positive drug test” [also known as an Adverse Analytical Finding (AAF)]. In recent years, analytical method sensitivity has improved to the point where laboratories are able to detect trace amounts of substances in a sample down to the parts per trillion (ppt) range.

Contamination of dietary supplements, foods, and compounded medications with substances prohibited in sport is common enough (reviewed in Gudeman et al., 2013; Martinez-Sanz et al., 2017; Tucker et al., 2018) that the anti-doping results management process for Olympic and Paralympic sport, as well as many professional sports, have changed their policies to account for such cases. In 2014 an athlete tested positive for the diuretic hydrochlorothiazide (HCTZ), whereupon investigation, was found the athlete had declared ibuprofen (a permitted medication) that was contaminated with ~2.5 mg of HCTZ per tablet (Helmlin et al., 2016). This example, in addition to the observations in the present manuscript, suggest that medications pose an emerging anti-doping risk.

Even though pharmaceutical manufacturing is governed by strict quality control criteria, contamination does occur. However, there have been very few reports of contamination with active pharmaceuticals. Cases of contamination with active pharmaceuticals, present a threat to the integrity, safety and efficacy of prescription medication as well as posing a unique challenge to athletes who are subject to sport drug testing. First, few people, including athletes, would consider the possibility that a medication could be contaminated. Second, unlike dietary supplements which are largely considered to be optional, medications are often required to treat legitimate injuries and medical conditions, and athletes cannot always avoid using them. Third, while athletes can be educated to avoid dietary supplements that make performance-enhancing claims, there are no obvious indicators on a medication that it may be contaminated. Athletes and Anti-doping Organizations (ADOs) alike must be able to trust government-approved medications if they complete the necessary due diligence to understand whether it is permitted or banned in sport. Differentiating between intentional doping (that is, athletes that purposely use a prohibited substance or method with the aim to enhance their performance) and product contamination through anti-doping sample analysis alone is challenging, and now compounded by the laboratories ability to detect trace amounts of prohibited substances. However, as stipulated in the global anti-doping rules, the burden remains on the athlete to prove the source of a positive test, which becomes unfair in cases of trace contamination where the source is not obvious or no longer available.

Here, we document nine instances of generically produced prescription medication, all contaminated with at least one diuretic (HCTZ, triamterene, and/or torasemide) which resulted in low level AAFs. The cases were identified through routine anti-doping processes conducted by USADA. A great deal of time, effort and resources were put into these cases to identify the source of the positive urine tests, with expeditious cooperation from all athletes involved, and it is probably unreasonable to expect that all ADOs, labs and athletes world-wide will have the resources to identify the source of contamination in every case. Here, we present data to support a minimum reporting level (MRL), below which labs would not be required to report findings, or report them as atypical. Implementing this MRL is a pragmatic solution that would effectively reduce the likelihood of unfairly sanctioning an athlete that has inadvertently consumed a medication contaminated with a diuretic.

## Methods and Results

Between 2017 and 2020, the Unites States Anti-Doping Agency (USADA) identified nine cases during the results management process where a positive anti-doping test could be accounted for by contamination in commercially manufactured, generic, prescription medication (Table 1).

**Table 1:** Summary of nine positive athlete samples at USADA where a generically manufactured prescription medication was found to be contaminated.

**Year**	**Sport**	Substance in athlete sample^*^	**Medication**	**Product lab testing results**
2017	Paralympic alpine ski	Torasemide (0.16 ng/ml)	Metformin 500 mg tablets	Torasemide 1.5 ng/tablet HCTZ 30 ng/tablet
2018	Swimming	HCTZ (0.5 ng/ml)	Cephalexin 500 mg capsules	HCTZ 30 ng/capsule in shell coating; HCTZ not detected in capsule contents
2018	Paralympic track and field	HCTZ (0.57 ng/ml)	Oxybutynin ER 15 mg tablets	HCTZ 540 ng/tablet
2020	Paralympic track and field	Torasemide (0.9 ng/ml)	Baclofen 30 mg tablets	Torasemide 0.4 ng/tablet
2020	Track and field	HCTZ (0.05 ng/ml)	Bupropion 100 mg tablets	Furosemide 6.8 and 7.5 ng/tablet HCTZ 14 and 17 ng/tablet (2 tablets tested)
2020	Archery	HCTZ (0.26 ng/ml)	Levothyroxine 100 mcg tablets	HCTZ 26–680 ng/tablet (3 tablets tested)
Redacted	Redacted	HCTZ (0.3 ng/ml)	Metformin 1,000 mg tablets	HCTZ 1 μg/ml
Redacted	Redacted	HCTZ (4 ng/ml) triamterene (0.3 ng/ml)	Nabumetone 750 mg tablets	HCTZ 640 ng/tablet Triamterene ND
Redacted	Redacted	HCTZ (1.7 ng/ml)	Zolpidem 5 mg tablets	HCTZ 80 ng/tablet

* *Estimated urinary concentration of the specific diuretic provided in parentheses*.

Urine samples were analyzed at a WADA accredited laboratory using standard in-or out-of-competition screens as appropriate. In all cases the B-sample analysis confirmed the A-sample finding and the results management process was initiated.

Following notification of the athlete of the positive result, tablets or capsules of the original prescription in their original packaging were obtained from the athlete in each case presented and forwarded to the WADA accredited laboratory for independent testing. The presence of the detected prohibited substance(s) was independently verified and confirmed using tablets or capsules remaining from the container and matched the declaration the athlete made at the time of doping control. In some cases, an independently sourced prescription from the same pharmacy and same medication was obtained, however due to rapid changes in lot number and pharmacy purchasing, it was not always possible to obtain the same lot number or manufacturer. Pictures of the medication bottles were obtained to verify the prescription, medication, and manufacturer, and extensive athlete interviews and follow-up testing conducted to rule out manipulation. All the athletes received no fault findings after an extensive investigation into the circumstances of the positive test.

Analysis of the medications was undertaken for representative samples. Extractions were made of entire dosage units including capsule shells where applicable. Extracts were prepared using methanol as solvent. Primary extracts were processed using polymeric reversed-phase solid-phase extraction cartridges incorporating wash steps to remove highly polar components. The extracted samples were analyzed by LC-MS/MS using accurate mass LC-MS/MS on a Q-Exactive Plus (Thermo Scientific) mass spectrometer with Orbitrap analyzer and a HESI-II ionization interface coupled to an UltiMate 3000 (Thermo Scientific) UHPLC system. Although product testing does not fall under the scope of WADA accreditation, compound identities were established using methods and identification criteria consistent with WADA requirements (WADA Technical document TD2015IDCR-“Minimum Criteria for Chromatographic-Mass Spectrometric Confirmation of the Identity of Analytes for Doping Control Purposes”) Sample chain of custody was likewise maintained following WADA guidelines (WADA Technical document TD2009LCOC-“Laboratory Internal Chain of Custody”).

In each case, the medication the athlete was using was a permitted over-the-counter or prescription medication, and the contaminating substance was either a single, or multiple diuretics. The most common contaminant observed was HCTZ (8 instances), followed by torasemide (2 instances) and triamterene (1 instance). In each case, the medications were prescribed by the athlete's health care provider and were declared by the athlete.

There was no common factor between the contaminated medications other than the contaminating substance was a diuretic and the medications were generic. Two of the medications were marketed by the same company, but the remaining seven medications were all marketed by different companies (data not shown). The medications were purchased in nine different states in the US by different retail pharmacies. Due to the complexities of obtaining accurate information and no reported adverse health events, the medications were not traced back to the original manufacturing plant or country of origin.

The amount of contaminating substance detected in the medications ranged from the tens of nanograms up to 1 mg (Table 1). The contamination was found in the contents portion of the medication, as opposed to the coating or capsule shell, in all but one case which suggests the contamination occurred during the manufacturing process and not at the dispensing pharmacy. The dosage of diuretics delivered in the contaminated medications ranged from tens of thousands to hundreds of thousand-fold *lower* than the typical therapeutic dosages which range from 12 to 200 mg for HCTZ, 5–200 mg for torasemide, and 100–200 mg for triamterene. The estimated urinary concentrations in the anti-doping tests associated with these contamination cases were all below 5 ng/ml with most being below 1 ng/ml. Urinary metabolites of the parent compounds, such as chlorothiazide, were not detected in any of the cases. Although there is a high degree of inter-individual variability, and the observed urinary concentration in a given sample varies with the pharmacokinetic and pharmacodynamic properties of the substance ingested and time of sample collection, it is clear that the concentrations observed in the urine due to contamination are multiple orders of magnitude below what would be considered a recently ingested dose that has a performance-enhancing effect.

There was also no common factor regarding the athletes or sports involved in the cases. The athletes were members of various sports (Table 1) including alpine skiing, swimming, track and field and archery. None of the cases reported here involved athletes in sports with weight categories which are traditionally considered at-risk for abuse of diuretics for weight-loss.

**Figure 1:**
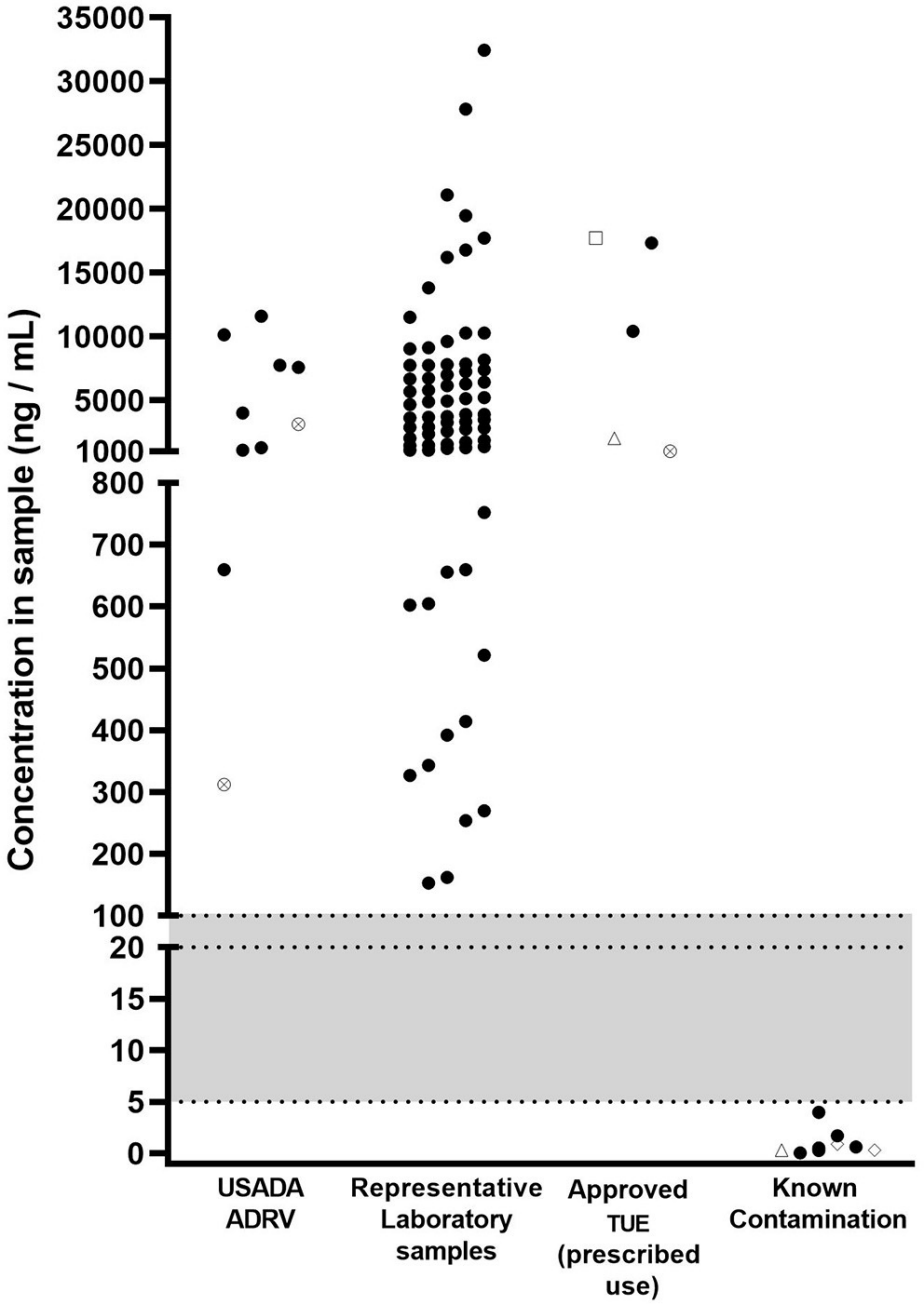
Concentrations of substances [HCTZ (black circles), furosemide (crossed circles), torasemide (open diamonds), triamterene (open triangles), acetazolamide (open square)] in samples returning adverse analytical findings for S.5 (diuretics and other masking agents) between 2017 and 2020. USADA ADRV: refers to samples resulting in an anti-doping rule violation (ADRV); Representative laboratory samples: refers to sample data provided by a laboratory returning Adverse Analytical Findings (AAFs) for HCTZ; Approved TUE (prescribed use): USADA samples where results management determined that the athlete had an approved TUE at the time of sample collection; Known contamination: refers to samples described in the present paper where contaminated medication has been identified as the source. Shaded area indicates a potential range of minimum reporting limit values.

In an attempt to delineate legitimate use of prescription diuretics from accidental contamination, we plotted the urinary concentrations from anti-doping tests from athletes that received anti-doping rule violations or had Therapeutic Use Exemptions (TUEs) with the urinary concentrations observed in the nine confirmed contamination cases (Figure 1). Also plotted is a much larger dataset from a World Anti-doping Agency (WADA) accredited lab reporting AAFs for HCTZ (Figure 1). The gray part of the figure shows a potential range for a MRL. Figure 1 shows that a range of 5–100 ng/ml is a reasonable range for a MRL that separates intentional use from contamination in the nine cases we report here.

## Conclusions and Recommendations

The cases described here demonstrate that prescription medications may be contaminated at levels high enough to cause a positive anti-doping test. In anti-doping drug tests, diuretics are considered non-threshold substances therefore detection at any level in an athlete's urine sample is deemed an AAF. Under the World Anti-Doping Code, athletes are responsible for all prohibited substances found in their biological samples collected for the purposes of doping control, and they bear the burden for demonstrating the source of any AAF. Even though diuretics are “specified substances” and there is some flexibility of sanction if it is established that athlete had no fault or negligence, the burden of proof remains on the athlete. However, athletes do not have readily available access to the kind of analytical testing that would support a claim of product contamination since external laboratories that test to the trace levels required in anti-doping are few and far between. Relying on the results management process within the ADO is also not currently feasible since not all ADOs have the resources to conduct in-depth investigations, or are aware that the level detected in the athlete sample was in fact extremely low.

Contamination of prescription medication is not limited to US athletes participating in Olympic and Paralympic sports. Corroborating the experience of the cases run by the USADA, there is at least one other unpublished report (not included in Table 1) of a generically manufactured prescription drug contaminated with a diuretic in a professional sport in the US. In this case, analysis of the athlete's prescriptions for bupropion and levothyroxine showed the presence of HCTZ in both products at 5 mcg/tablet and 2 ng/tablet respectively (Eichner and Van Wagoner, unpublished observation). We are also aware of at least one other instance of a prescription medication contaminated with acetazolamide in Japan, in addition to the previously published account of a contaminated product in Switzerland from 2014 (Helmlin et al., 2016). These observations suggest this is a global concern for athletes in all sports.

**Figure 2:**
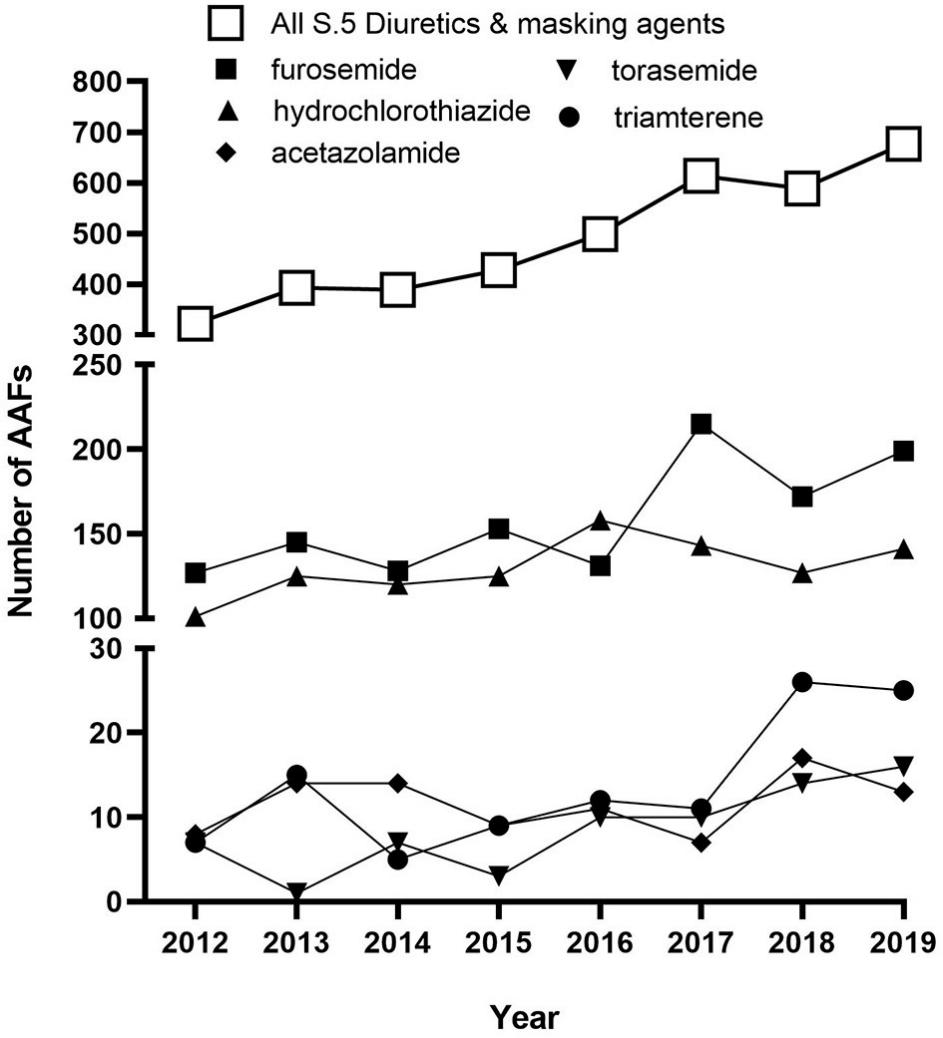
Historic global trends for AAFs for substances in the class of S5. Diuretics and masking agents (open squares) and particular diuretics (closed symbols) as reported in the 2012–2019 Anti-Doping Testing Figures.

The most frequent contaminant in the present study was HCTZ, which was involved in eight of the nine cases reported here (Table 1). Perhaps it is not surprising as HCTZ was the 13th most prescribed medications in the United States in 2018 with over 40 million prescriptions (ClinCalc.com)^1^. HCTZ is also one of the most frequently detected diuretics in international anti-doping samples. According to the 2019 WADA Testing Figures (WADA Agency, 2019) HCTZ accounted for almost a third of all positives for diuretics (677 total diuretic AAFs; furosemide 29%; HCTZ and metabolites (28%); canrenone (spironolactone metabolite) 10%; dorzolamide 7%), and the overall rate of positive tests for diuretics is increasing (Figure 2). Torasemide and triamterene are also frequently represented in the top 10 diuretics detected over the same time-period and typically account for 1–2% of detected diuretic samples. It is important to note that included in these AAFs are athletes that may have permission to use a diuretic for treatment of a legitimate medical condition through a TUE. As the level and source of diuretic detected in an athlete sample is not published in the WADA Testing Figures, it is unknown how many of the cases globally could be accounted for by contaminated medications.

In conclusion, it is clear that anti-doping has crossed a threshold where it is has shown that pharmaceutical contamination can account for at least some low-level positive anti-doping tests. Due to the resource-intensive investigations often required to identify the source of contamination, it is unlikely that all cases that occur globally will be identified during the results management process. Further, it is unfair and unethical to expect athletes to refuse the use of otherwise permitted medications out of fear of returning a positive test, or to sanction athletes for the inadvertent (but unidentified) use of a prohibited substance due to contamination. Therefore, the authors recommend implementing a MRL for specific diuretics based on prevalence of low-level findings. The observations in this study support a conservative MRL. A MRL between 5 and 100 ng/ml accurately discriminates between use of therapeutic doses vs. contamination in the nine real-world cases presented here. As a result of a global consensus, WADA announced in June 2021 a new Stakeholder Notice regarding potential diuretics contamination cases which implements a MRL at or below 20 ng/ml, with additional investigation provisions for weight category sports that helps to ensure cases of diuretic contamination are managed fairly for all athletes.

## Data Availability Statement

The data analyzed in this study is subject to the following licenses/restrictions: Data includes sensitive testing results which cannot be publicly shared. Requests to access these datasets should be directed to m.fedoruk@usada.org.

## Ethics Statement

Ethical review and approval was not required for the study on human participants in accordance with the local legislation and institutional requirements. Written informed consent for participation was not required for this study in accordance with the national legislation and the institutional requirements. Written informed consent was obtained from the individual(s) for the publication of any potentially identifiable images or data included in this article.

## Author Contributions

MF designed the work. LL, RV, DE, and MF provided analysis or interpretation of data for the work and revised it critically for important intellectual content. AE, LL, and BL drafted the work. All authors provided approval for publication of the content and agree to be accountable for all aspects of the work in ensuring that questions related to the accuracy or integrity of any part of the work are appropriately investigated and resolved.

## Conflict of Interest

The authors declare that the research was conducted in the absence of any commercial or financial relationships that could be construed as a potential conflict of interest.

## Publisher's Note

All claims expressed in this article are solely those of the authors and do not necessarily represent those of their affiliated organizations, or those of the publisher, the editors and the reviewers. Any product that may be evaluated in this article, or claim that may be made by its manufacturer, is not guaranteed or endorsed by the publisher.

## Footnotes

^1^ClinCalc Drug Stats Database.
